# Genetic Evidence of Hybridization between the Endangered Native Species *Iguana delicatissima* and the Invasive *Iguana iguana* (Reptilia, Iguanidae) in the Lesser Antilles: Management Implications

**DOI:** 10.1371/journal.pone.0127575

**Published:** 2015-06-05

**Authors:** Barbara Vuillaume, Victorien Valette, Olivier Lepais, Frédéric Grandjean, Michel Breuil

**Affiliations:** 1 Muséum national d’Histoire naturelle, Laboratoire des Reptiles et Amphibiens, Bâtiment 30, 57, rue Cuvier, CP n° 30, 75231, Paris, cedex 05, France; 2 Laboratoire Genindexe, 7 rue des Sports, 17000, La Rochelle, France; 3 Laboratoire Ecologie et Biologie des Interactions, équipe: Ecologie, Evolution, Symbiose, UMR CNRS 7267, Université de Poitiers, 6 rue Michel Brunet, 86022, Poitiers, Cedex, France; 4 INRA, UMR 1224, Ecologie Comportementale et Biologie des Populations de Poissons, Saint Pée sur Nivelle, France; 5 Univ. Pau & Pays Adour, UMR 1224, Ecologie Comportementale et Biologie des Populations de Poissons, Anglet, France; National Cheng-Kung University, TAIWAN

## Abstract

The worldwide increase of hybridization in different groups is thought to have become more important with the loss of isolating barriers and the introduction of invasive species. This phenomenon could result in the extinction of endemic species. This study aims at investigating the hybridization dynamics between the endemic and threatened Lesser Antillean iguana (*Iguana delicatissima*) and the invasive common green iguana (*Iguana iguana*) in the Lesser Antilles, as well as assessing the impact of interspecific hybridization on the decline of *I*. *delicatissima*. 59 *I*. *delicatissima* (5 localities), 47 *I*. *iguana* (12 localities) and 27 hybrids (5 localities), who were all identified based on morphological characters, have been genotyped at 15 microsatellites markers. We also sequenced hybrids using ND4 mitochondrial loci to further investigate mitochondrial introgression. The genetic clustering of species and hybrid genetic assignment were performed using a comparative approach, through the implementation of a Discriminant Analysis of Principal Component (DAPC) based on statistics, as well as genetic clustering approaches based on the genetic models of several populations (Structure, NewHybrids and HIest), in order to get full characterization of hybridization patterns and introgression dynamics across the islands. The iguanas identified as hybrids in the wild, thanks to morphological analysis, were all genetically F1, F2, or backcrosses. A high proportion of individuals were also the result of a longer-term admixture. The absence of reproductive barriers between species leads to hybridization when species are in contact. Yet morphological and behavioral differences between species could explain why males *I*. *iguana* may dominate *I*. *delicatissima*, thus resulting in short-term species displacement and extinction by hybridization and recurrent introgression from *I*. *iguana* toward *I*. *delicatissima*. As a consequence, *I*. *delicatissima* gets eliminated through introgression, as observed in recent population history over several islands. These results have profound implications for species management of the endangered *I*. *delicatissima* and practical conservation recommendations are being discussed in the light of these findings.

## Introduction

Hybridization between two closely related species is a natural phenomenon observed in around 10% of animals and 25% of plant species [1]. Hybridization brings novelty in a gene pool, thus increasing fitness in new environments, and even speciation [2]. It requires gene flow and can occur when species are found in sympatry and when reproductive isolation is incomplete [3]. With the loss of isolating barriers, due to anthropogenic factors and the introduction of invasive species, hybridization has become a threat to biodiversity, as endangered species could be lost in the case of intensive hybridization with congeners from exotic species [4]. Numerous examples [5] of the harmful effects of hybridization in plant and animal taxa have been known to yield to extinction, with or without introgression. Several cases of hybridizations have been reported within and between different genera in iguanas [6, 7]. Gutsche & Köhler [8, 9] demonstrated that *Ctenosaura similis*, a coastal species, hybridizes with the insular *C*. *bakeri* and poses serious conservation issues for this latter.

The Lesser Antillean Iguana (*Iguana delicatissima*) is an endemic species found from Martinique to Anguilla [4, 10]. In some islands, high hunting pressure and the rapid transformation of the littoral for housing, accommodation, and agriculture, have led to a decline of this species throughout its range [11, 12, 13, 14]. Introduced predators such as rats, cats, dogs, and raccoons, in Guadeloupe, and possums in Martinique [13, 15, 16], have certainly contributed to this decline. Road traffic must be an important factor, as in La Désirade, where hundreds of *I*. *delicatissima* may be killed each year (Breuil, personal obs., April 2009), as well as in St. Barthélemy (Breuil, personal observation, April and July 2011).

Historically, *I*. *delicatissima* is present on all islands from Martinique to Anguilla, except on Montserrat and Saba, which are inhabited by the green iguana, *I*. *iguana*. The green iguana *I*. *iguana* is native to Latin America, including parts of Mexico, as well as the mainland and island regions of Central and South America. Phylogenetic works showed that *I*. *iguana* from Montserrat and Saba is very close genetically and belongs to a South American lineage (from Venezuela & Suriname), which is much different from the Central America lineage [17, 18]. *I*. *iguana* was unknown in Guadeloupe at the time of colonization, as shown by all the chronicles and samples collected by old naturalists [4, 12, 19]. However, Grouard *et al*. [20] found *I*. *iguana* and *I*. *delicatissima* remains in the refuse middens in Basse-Terre and Grande-Terre (Guadeloupe) during the Saladoid period (500 AD). It is impossible to know whether those common iguanas were hunted on these islands or if they came from other islands.

Breuil [21] proposed that *I*. *iguana* had been arriving in Les Saintes from French Guyana since the middle of the 19^th^ century, probably as stowaways on prison boats circulating between these two places. The first *I*. *iguana* were caught in Les Saintes in 1914 by Noble. They are preserved in the Museum of Comparative Zoology. At that time, hybridization had already begun with *I*. *delicatissima* [21]. According to Lazell [22], in the 1960s, both species were present in this archipelago. In the 1990s, *I*. *delicatissima* was very rare and hybrids were present [23]. At the beginning of the 21^st^ century, it was impossible to find a pure *I*. *delicatissima* in Les Saintes. For example, *I*. *delicatissima* was present in the 1960s in Terre-de-Bas des Saintes [22], but it had been replaced by *I*. *iguana* and hybrids at the beginning of the 1990s [12]. This situation clearly shows that this allochthonous *I*. *iguana* competes with *I*. *delicatissima*, yielding to its extinction. This phenomenon is currently in progress in Basse-Terre and St. Barthélemy.

The work of Lazell [22] was regarded as a good description of the situation in the French West Indies (FWI), where there was no competition between the two species [19]. Thus, both iguana species have been protected in Guadeloupe since 1989, whereas *I*. *iguana* has not been protected in Martinique because it was known that it was introduced there [15, 24]. At the beginning of the 1990s, morphological and genetic analyses of these mixed populations demonstrated, beyond any doubt, the hybridization between the two species [25].

Based on the situation in the 1990s, the Iguana specialist (ISG/IUCN) group first proposed to classify *I*. *delicatissima* as “vulnerable” [11].Ten years later, this species was upgraded to “in danger” because of the situation in FWI where *I*. *delicatissima* disappeared from Les Saintes (Terre-de-Haut, Terre-de Bas), St. Maarten-Martin [26, 27], Grande-Terre, and satellites of St. Barthélemy [14, 28]. Some *I*. *delicatissima* are still present in Basse-Terre. However its last pure populations, which were known in the mid-1990s [12], were invaded by *I*. *iguana*, which lead to the production of individuals with intermediate phenotypes [29]. Lorvelec & Pavis [30] rejected, without any argument, competition and hybridization as the main cause for the disappearance of *I*. *delicatissima*. Local authorities in charge of nature conservation followed their position and nothing was done in Guadeloupe to prevent both the extension and hybridization of *I*. *iguana* with *I*. *delicatissima*. This has led to the disappearance of all breeding populations from the Lesser Antillean Iguana through competition and hybridization in Grande-Terre and Basse-Terre. In this context, the paper of Lorvelec *et al* [31] mentioning the presence of *I*. *delicatissima*, without any information on these islands, is misleading. Those authors were greatly confused between the persistence of some individuals and the presence of functional breeding populations of this species giving birth to pure *I*. *delicatissima* offspring [32, 33].

From the present study, we address the following questions regarding *I*. *delicatissima* and *I*. *iguana*: (1) Is there any evidence of hybridization between these species by using bi-parentally inherited microsatellites? (2) Are the hybrids able to reproduce? (3) Does hybridization occur in both directions through analysis of mtDNA? (4) What are the implications of these results in terms of conservation requirements?

## Material and Methods

### Populations sampling and DNA isolation

The DREAL (Direction Régionale de l’Environnement, de l’Aménagement et du Logement), the relevant office concerned with protection of Wildlife in French Caribbean islands, validated the sampling protocol and gave their authorization for this study. All sampling locations are available in Table 1. All sampling locations were private. Authors confirmed that they were granted permission from all landowners to access the land and conduct the study. All biopsies were made by French veterinary services, including the following veterinaries: Béatrice Ibéné (Guadeloupe), Chloé Rodrigues (Martinique), and Jean-Claude Mailles (St. Barthélemy and North islands).

**Table 1:** Origins of studied samples, grouped based on their putative identification in the field. Localities are grouped by islands and from North to South. Numbers in brackets correspond to specimen analyzed using mtDNA.

	Location	Bank	Coordinates	Nb Ind.
***I*. *delicatissima***	St. Barthélemy (St. Jean)	Anguilla	62°50'23.06"W; 17°54'5.78"N	3 (3)
	Basse-Terre (Cluny)	Guadeloupe	61°44'56.03"W; 16°21'15.26"N	1 (1)
	Basse-Terre (Carangaise-Longueteau)	Guadeloupe	61°34'18.13"W; 16° 4'49.73"N	11 (5)
	Petite-Terre (Terre de Haut)	Guadeloupe	61°06' 40,4''7W; 16°10 38,07''N	15 (5)
	Chancel	Martinique	60°53'22,35''W; 14°4137.52''	29 (6)
***I*. *iguana***	Saint-Martin (Anse Marcel)	Anguilla	63°2'28.18"W; 18° 6'41.65"N	4
	Saba	Saba	63°13'46.25"W; 17°38'27.25"N	7 (4)
	Basse-Terre (Carangaise-Longueteau)	Guadeloupe	61°34'18.13"W; 16° 4'49.73"N	4
	Grande-Terre (St. François-Manganao)	Guadeloupe	61°17'36.08''W; 16°14'41,92''N	2 (1)
	Grande-Terre (Gosier)	Guadeloupe	61°29'8.95"W; 16°12'18.32"	5 (5)
	Terre-de-Haut	Saintes	61°34'47.19"W; 15°52'1.07"N	5
	Fort-de-France	Martinique	61° 3'57.94"W; 14°36'11.72"N	3 (3)
	St. Lucia	St. Lucia	60°53'29.09"W; 14°1'5.62"N	13 (4)
	Central America (Salvador)	From breeding farm	88°48'27.59"W; 13°50'50.81"N	1 (1)
	South America (Venezuela, Rio Caroni)		?	1
	South America (French Guyana, Yalimapo)		53°55'56.66"W; 5°41'27.77"N	1
	South America (French Guyana, Camopi)		52°20'27.96"W; 3° 9'55.89"N	1
**Hybrids**	Saint Barthélemy (St. Jean)	Anguilla	62°50'23.06"W; 17°54'5.78"N	2 (1)
	Basse-Terre (Cluny)	Guadeloupe	61°44'56.03"W; 16°21'15.26"N	6 (5)
	Basse-Terre (Anse à sable)	Guadeloupe	61°46'29.17"W; 16° 9'2.34"N	3 (2)
	Basse-Terre (Carangaise-Longueteau)	Guadeloupe	61°34'18.13"W; 16° 4'49.73"N	5 (3)
	Grande-Terre (St François-Manganao)	Guadeloupe	61°17'36.08''W; 16°14'41,92''N	11 (7)

DNA was obtained from animals caught in the field either using nooses (the majority) or by hand. For each iguana, the tip of the tail was cut with a sterile scalpel and disinfected. The biopsy was opened to facilitate ethanol penetration in the tissues and stored in 70% ethanol according to methods used by iguana specialists. The morphology of the iguana was described using the characters depicted by Breuil [12, 21] and pictures were taken. Each animal has a Passive Integrated Transponder (PIT) tag in its left leg, with an identification number for conservation purposes (see below).

**Fig 1:**
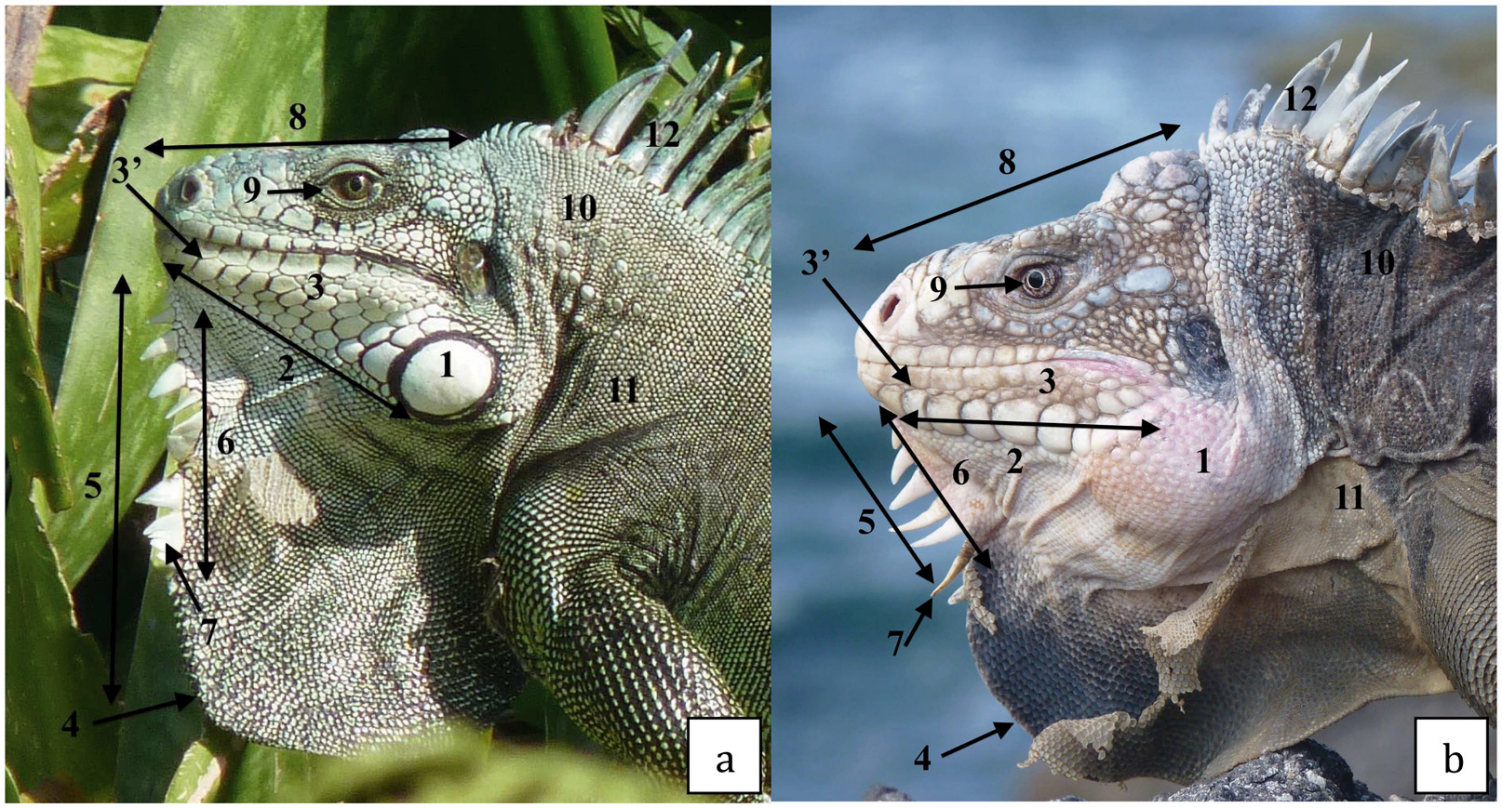
Main head morphological differences between the two species. Morphological characterisation of the head of *Iguana iguana* (Fort Saint-Louis, Martinique). 1. Subtympanic plate. 2. Lower sublabial scales forming a mosaic. 3. Flat sub-labial and labials. 3’. No row of oval scales between the labials and sub-labials. 4. Dewlap edges forming nearly a right angle. 5. Gular spikes extending into the lower half of the dewlap. 6. Gular spikes > 10. 7. Triangular gular spikes. 8. Top of head flat. 9. Eye chestnut brown. 10. Nuchal tubercles. 11. Body colour greenish grey. 12. High dorsal crest scales. Morphological characterization of the head *Iguana delicatissima* (St. Barth). 1.No subtympanic plate. 2. Sublabial row of scales ± parallel to the labial scales. 3. Rounded scales. 3’. Row of oval scales between the labials and sub-labials. 4. Rounded dewlap edge. 5. Gular spikes on the straight upper edge of the dewlap. 6. Gular spikes <7–8. 7. Conical, long and more or less curved gular spikes. 8. Top of head bumpy. 8’. Occipital bumps. 9. Grey eye. 10. Lack of nuchal tubercles. 11. Body colour brownish-grey. 12. Moderately high dorsal crest scales.

**Fig 2:**
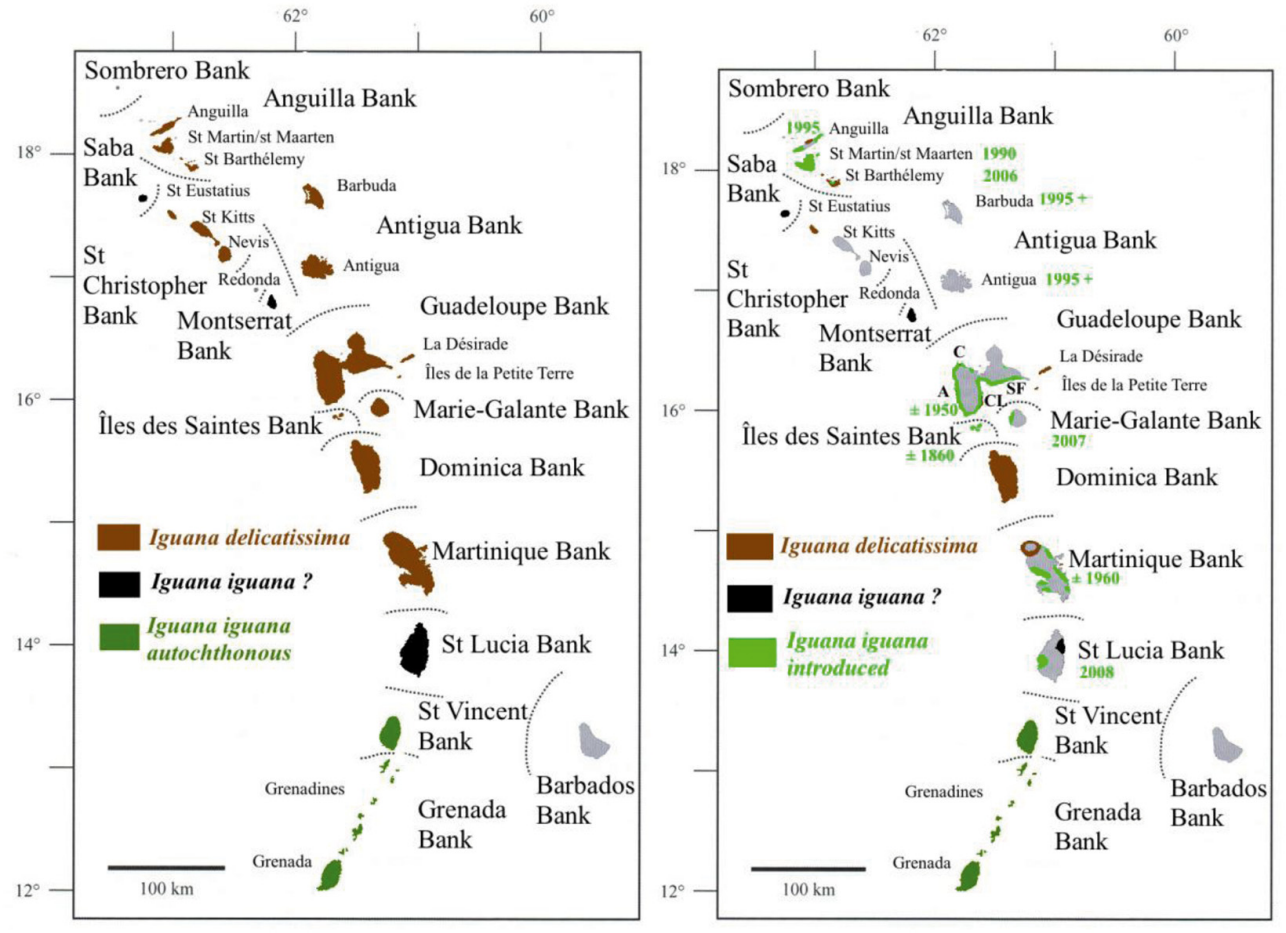
Maps of the distribution of the two iguana species in Lesser Antilles. The left map represents the historical distribution of the species as far it was in the Sixties. The right map is the today distribution. *Iguana iguana*? represents local populations of uncertain status which are morphologically distinct from continental iguanas (Breuil, 2013). Since then seven main islands lost their *Iguana delicatisisma* between the Sixties and the Nineties. The dates in green indicate when the allochthonous *I*. *iguana* from South and Central America arrived. For Guadeloupe Bank, **C** indicates the locality of Cluny, **A** the locality of Anse à Sable, **CL** the locality of Carangaise-Longueteau and **SF** the locality of Saint-François. All these latter localities are inhabited by hybrid populations where *I*. *iguana*, *I*. *delicatissima* and hybrids are still present.

A sampling of 133 individuals was analyzed with 59 *I*. *delicatissima*, 47 *I*. *iguana*, and 27 individuals considered as hybrids according to morphological criteria defined by Breuil [21]. The main morphological differences between the heads of both species are presented in Fig 1. Other differences exist, such as the plain tail of *I*. *iguana* compared with the banded tail of *I*. *delicatissima*, or the green coloration of *I*. *iguana* compared with the brown coloration of *I*. *delicatissima*. Geographical distribution of both species in the Caribbean islands, as well as information concerning the dates of arrival of the invasive *I*. *iguana*, are represented in Fig 2. *I*. *delicatissima* individuals from Chancel (Martinique) and Terre de Haut (Petite Terre) were from populations in which no *I*. *iguana* were observed or were known to have lived. All hybrids come from mixed populations where both species are actually present [24] and have been identified by more than 15 morphological characters [21] from two different populations in Basse-Terre, one in Grande-Terre and one in St. Barthélemy.

**Fig 3:**
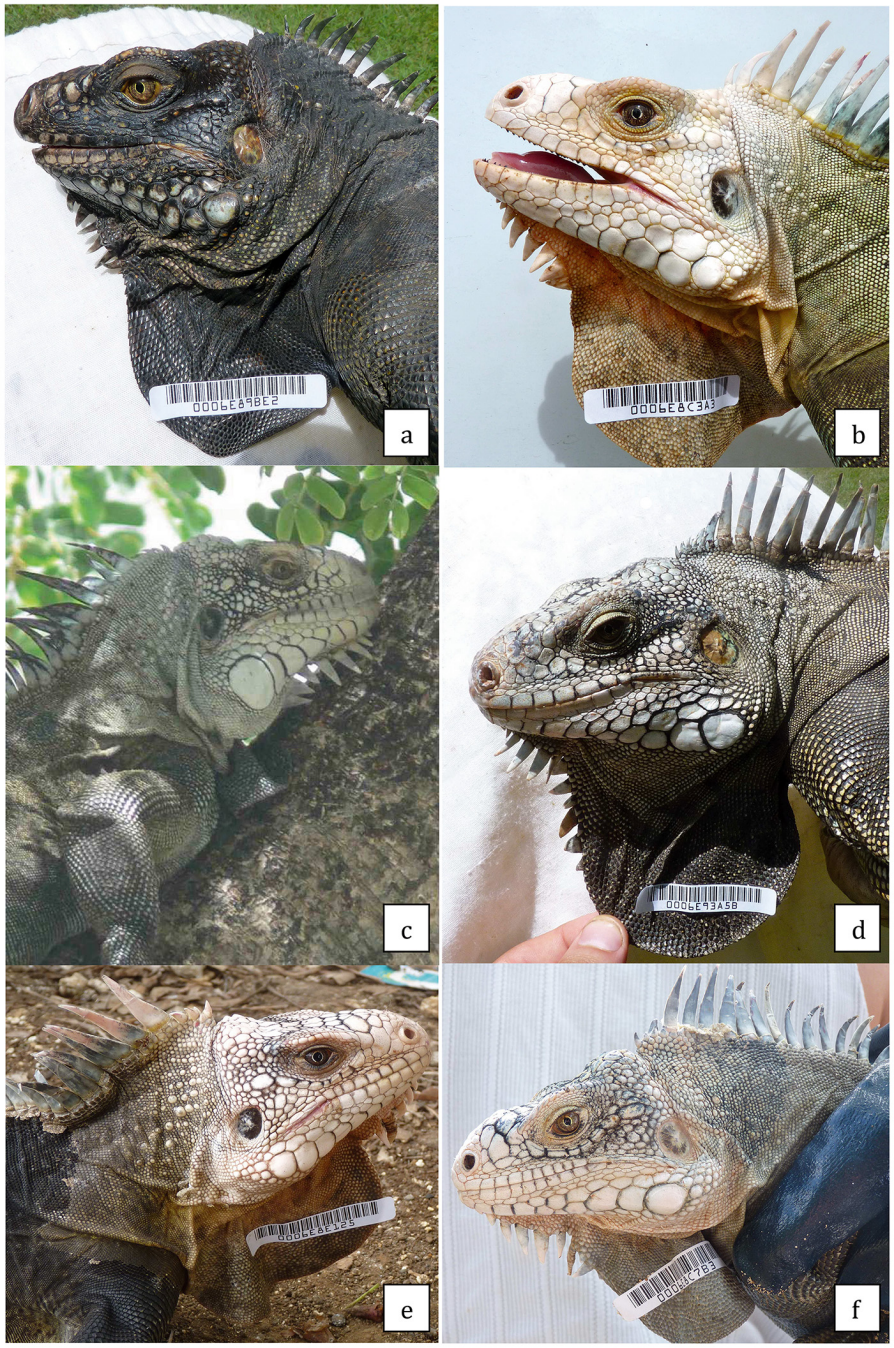
Photos of different categories of hybrids. Hybrid (Basse-Terre, Guadeloupe) (IGH 49): probability backcross *delicatissima*, 0.98; probability F2, 0.02 (a) Hybrid (Grande-Terre, Guadeloupe) (IGH 39): probability F2, 0.99; probability backcross *delicatissima*, 0.01(b). Hybrid Grande-Terre (Guadeloupe) (IGH 9): probability F1, 0.80; probability F2, 0.06; probability backcross *delicatissima*, 0.07; probability backcross *iguana*, 0.07. (c) Hybrid Grande-Terre (Guadeloupe) (IGH 43): probability backcross *iguana*, 0.86; probability *iguana* 0.14 (d). Hybrid (Basse-Terre, Guadeloupe) (IGH 48): probability F2, 0.60; probability backcross *delicatissima*, 0.40 (e). Hybrid (Basse-Terre, Guadeloupe) (IGH 44): probability backcross *delicatissima*, 0.83; probability F2, 0.17 (f).

The hybrid status of an iguana is defined in the field with respect to the following conditions: both parental species were observed during the fieldwork according to diagnostic characters depicted in Fig 1 and individuals that present various intermediate conditions, for the whole set of characters and/or exhibit a mosaic of both parental or intermediate characters, are considered as hybrids. Table 2 gives some characters that, according to Breuil [12], were used to determine the hybrid condition of individuals. Only three states were recognized for each character: *iguana*, *delicatissima*, and intermediate. The intermediate state can range from nearly *iguana* to nearly *delicatissima*. Gular spikes and the subtympanic area regroup complex situations. The “Gular spikes” refer to their number, morphology, and position. Different kinds of hybrids (F1, F2, and backcrossed individuals with both parental species) are presented in Fig 3 with their probability to belong to each hybrid category as determined by Hliest (Table 2).

**Table 2:** Morphological characteristics of hybrids with their probability to belong to each hybrid category as determine by NewHybrids and their mitochondrial DNA haplotype (mitotype IGI: *I*. *iguana*; IGD: *I*. *delicatissima*).

		Newhybrids						Mitotype	Morphological chracteristics			
Hybrids code	Origin	*I*. *del*.	*I*. *igu*.	F1	F2	Bc1	Bc2	ND4	Subtympanic area	Gular spikes	Body color	tail
IGH37	StBarthélemy	0.00	0.00	0.00	0.09	**0.91**	0.00	IGI	int	Int	int	int
IGH38	StBarthélemy	0.00	0.00	0.00	0.09	**0.91**	0.00	-	int	Int	int	int
IGH8	Manganao	0.00	0.00	0.00	**0.73**	0.00	**0.27**	IGD	int	*iguana*	int	int
IGH9	Manganao	0.00	0.00	**0.80**	0.06	0.06	0.07	IGD	int	Int	int	int
IGH10	Manganao	0.00	**0.88**	0.00	0.00	0.00	0.12	IGI	*iguana*	Int	*iguana*	*iguana*
IGH11	Manganao	0.00	0.00	0.00	0.03	0.00	**0.97**	IGI	int	*iguana*	int	*iguana*
IGH12	Manganao	0.00	**0.26**	0.00	0.00	0.00	**0.74**	IGD	*iguana*	*iguana*	int	*iguana*
IGH14	Manganao	0.00	0.00	0.00	**1.00**	0.00	0.00	IGD	int	int	int	*iguana*
IGH39	Manganao	0.00	0.00	0.00	**0.99**	0.00	0.00	-	Int	int	int	int
IGH40	Manganao	0.00	0.00	0.00	0.01	0.00	**0.99**	IGD	int	int	int	*iguana*
IGH41	Manganao	0.00	0.00	0.00	0.00	0.00	**1.00**	-	int	*iguana*	*iguana*	*iguana*
IGH42	Manganao	0.00	0.00	0.00	0.01	0.00	**0.99**	-	int	*iguana*	*iguana*	int
IGH43	Manganao	0.00	0.13	0.00	0.00	0.00	**0.86**	-	int	int	*iguana*	*iguana*
IGH3	Cluny	0.00	0.00	0.00	**0.47**	0.00	**0.53**	IGD	int	int	*delicatissima*	*delicatissima*
IGH4	Cluny	0.00	0.00	0.00	**0.51**	**0.48**	0.00	IGD	int	int	int	int
IGH6	Cluny	**0.97**	0.00	0.00	0.00	0.03	0.00	IGD	*delicatissima*	*delicatissima*	*delicatissima*	*delicatissima*
IGH7	Cluny	0.00	0.00	0.00	0.18	0.00	**0.82**	IGI	int	*iguana*	int	int
IGH44	Cluny	0.00	0.00	0.00	0.17	**0.83**	0.00	IGD	int	*delicatissima*	int	int
IGH46	Cluny	0.00	0.00	0.00	0.03	0.00	**0.97**	-	int	*iguana*	*iguana*	*iguana*
IGH47	Carangaise	0.00	0.00	0.00	0.03	0.00	**0.97**	IGI	int	*iguana*	*delicatissima*	*iguana*
IGH48	Carangaise	0.00	0.00	0.00	**0.60**	**0.40**	0.00	-	int	*delicatissima*	*delicatissima*	*delicatisisma*
IGH49	Carangaise	0.00	0.00	0.00	0.02	**0.98**	0.00	IGD	int	int	int	*delicatissima*
IGH50	Carangaise	0.00	0.00	0.00	0.00	0.00	**1.00**	IGI	*iguana*	*iguana*	int	int
IGH52	Longueteau	0.00	0.00	0.00	0.01	0.00	**0.99**	-	*iguana*	int	int	int
IGH53	UCPAAnseASable	0.00	0.00	0.00	0.02	**0.98**	0.00	-	int	*delicatissima*	int	*iguana*
IGH1	GuadBTUCPA	0.00	0.00	0.00	0.02	0.00	**0.98**	IGI	int	*iguana*	int	*delicatissima*
IGH2	GuadBTUCPA	0.00	0.00	0.00	0.26	0.00	**0.74**	IGI	*iguana*	int	int	*delicatissima*

Int corresponds to intermediate morph.

The common iguanas are from different origins. Saba and St. Lucia iguanas have their own specific morphology and are considered endemic lineages [12, 21]. Iguanas from Central America and from South America are used because they come from natural distributions of *I*. *iguana*. These continental iguanas represent both lineages found by Stephen *et al*. [18]. The insular common iguanas from the French West Indies are from localities where *I*. *delicatissima* were either eliminated (St. Maarten-Martin, Les Saintes, Grande-Terre, and Martinique) in a recent past or are still present, such as in St. Barthélemy and Basse-Terre.

### Microsatellites analyses

We used 15 microsatellites amplification in both species [34]. All experimental procedures for microsatellite genotyping were strictly identical to those described in Valette *et al*. [34].

The average number of alleles per locus (A), the observed and expected heterozygosity (respectively H_O_ and H_E_) were computed with GENETIX 4.05 [35] and estimated for each species and the hybrids. Deviations from Hardy—Weinberg equilibrium were tested using the exact probability test of Guo & Thompson [36] available in GENEPOP 3.2a [37]. Significance levels were calculated at each locus and over all loci for each group. Genotypic linkage disequilibrium between each pair of loci was estimated by Fisher’s exact tests with GENEPOP 3.2a software. Both tests for deviations from Hardy—Weinberg equilibrium and for linkage disequilibrium used a Markov chain (1 000 dememorization steps, 100 batches, 1 000 iterations).

To characterize mitochondrial introgression, the NADH deshydrogenase subunit 4 gene (ND4) was amplified using the primers and PCR protocols in Malone *et al*. [38]. 20 individuals from *I*. *delicatissima*, 18 from *I*. *iguana* and 18 hybrids were analyzed (Table 1).

### Species genetic clustering

We used Structure version 2.3.4 [39, 40, 41] to assign individuals to species, using the genotype of 15 microsatellites for 133 individuals with no a priori assumption. We first assumed two species, therefore setting the number of clusters to two (K = 2), and made 10 independent runs using a burn-in period of 100,000 steps, followed by 200,000 Markov Chain Monte Carlo iterations, with an Admixture model of ancestry that allows for an individual to have mixed ancestry, as well as a Correlated Allele Frequency model, which assumes that the species underwent independent drift away from a hypothetical ancestral species. After ensuring that consistent results were obtained for each repeated analysis, the most likely of the 10 runs, which was indicated by the higher log likelihood value, was chosen for individual assignment to species and identification of putative hybrids. Estimated allele frequencies of each cluster (each corresponding to one species) were recorded for subsequent analyses.

### Hybrids genetic assignment

#### NewHybrids

We used NewHybrids version 1.1Beta3 [42] to assign individuals to a species or to one of the different hybrid classes: F1, F2, and backcrosses. The analysis was performed with the genetic data collected from 15 microsatellites genotyped in 133 individuals. We used Jeffrey's prior for both the allele frequencies and the mixing proportions. We ran 10,000 iterations as a burn-in period, followed by 20,000 MCMC repetitions. To assess our power to assign individuals, we used two different assignment strategies with different stringency criteria. In the first one (relaxed criteria), assignments were made to the most likely species or hybrid class. In the second one (strict criteria), assignment was made only for the individual with a probability higher than 0.90 to belong to one of the species or hybrid classes.

#### HIest

In addition to estimating admixture coefficient to assign individual to species (Structure and NewHybrids methods), we used the R package HIest [43], which enables us to conjointly estimate the ancestry coefficient (S; equivalent to admixture coefficient Q in Structure) and interclass heterozygosity (H_I_). Joint estimation of these parameters allows assessment of whether the studied hybrid system fits a simple two-generation hybridization model, as assumed by a classification method that relies on a restricted set of classes produced by two generations of hybridization (e.g. NewHybrids). This method assumed that allele frequencies of the hybridizing species were known, so we used cluster allele frequencies that had been previously estimated by Structure for K = 2 as an input for HIest. We ran HIest package function *HIest* into R version 2.15.2 [44] with simulated annealing algorithm as an optimization method to search for maximum likelihood estimates of ancestry and heterozygosity. Starting values were initiated by evaluating likelihoods on a grid of size 20, corresponding to 200 combinations of S and H_I_ values followed by 10,000 iterations, so as to obtain the joint posterior estimates of ancestry and interclass heterozygosity. We used function *HIclass* to calculate likelihoods for each of the six possible genotype classes in two generations of hybridization (each parental species, F1, F2, and backcrosses, toward each parental species). We finally compared the likelihood of hybrid classification (6 classes) to the maximum likelihood estimates of ancestry and interclass heterozygosity (continuous model of hybridization). To that end, we used function *HItest*, which has allowed us to decide whether the simple classification assuming an early hybridization system is acceptable if its AIC (Akaike Information Criterion) is lower than the AIC of the S and HI maximum log-likelihood estimates. This allowed us to estimate the age of interspecific crossing, given that a recent hybridization, or one limited to two generations by some biological constrains, would fit into a simple classification model, whereas a longstanding hybridization and introgression would not be supported by the simple classification. In a second phase, if the simple classification model had been accepted, we assessed the power for individual assignment by comparing the log likelihood of the best-fit class and the second best-fit class and accepted the assignment only if the best class was supported by more than 2 log-likelihood units (i.e. 95% confidence interval) over the second best class. Individual assignment to a specific hybrid class was then performed when the simple classification model fit the data and if the most likely genotypic class was significantly more supported than the other classes.

### Islands population genetic structure

We used a Discriminant Analysis of Principal Component (DAPC) [45], implemented in the *adegenet* package [46] available for R version 2.15.2 [44], to investigate the population genetic structure between species and between populations within species. It has been proven that this multivariate approach is well suited to identify complex genetic structures, such as the hierarchical population structure [47], which can be expected in the studied system with relationship both at the species (species and hybrids) and population levels (islands). In this situation, application of the hierarchical Evanno method [48] to determine the best number of clusters would be difficult as the presence of admixed individuals (hybrids) at the uppermost hierarchical population genetic structure level (species level at K = 2) would be removed from the analysis, in order to identify clusters within species.

We ran DAPC by first performing a principal component analysis (PCA) to transform the raw genetic data, thus retaining all principal components to maximize the variation of the original data. K-means clustering was applied using the function *find*.*clusters* to identify the best number of clusters K that minimize the variation within clusters using Bayesian information Criterion (BIC). We assumed a maximum number of 40 clusters and ran the K-means algorithm with 1,000 random starting values and 100 000 000 iterations to insure convergence. We chose the best number of cluster K as the one that showed the smallest BIC value. A discriminant analysis (DA) was then applied with the *dapc* function using 30 PCs, explaining more than 85% of the total variance of the data and retaining 7 discriminant functions that carried most information. Membership probabilities of each individual to each cluster was represented at the individual and population levels and then summed up across species and hybrids. Relationships between individuals and clusters in the DAPC space were visualized using respectively a scatterplot and a neighbor-joining tree (R package *ape*) [48], which were computed based on the DAPC distance between centroids of the different clusters.

### Mitochondrial Introgression in hybrids

To characterize mitochondrial introgression, the NADH deshydrogenase subunit 4 gene (ND4) was amplified using the primers and PCR protocols in Malone *et al*. [38]. A total of 20 individuals from *I*. *delicatissima*, 18 from *I*. *iguana*, and 18 hybrids have been analyzed (Table 1).

## Results

### Genetic diversity

Among the 15 microsatellites loci examined, 12, 15, and 14 are polymorphic respectively for *I*. *delicatissima*, *I*. *iguana* and hybrids (S1 Table). There was no evidence of linkage disequilibrium between loci. Allelic richness was 2.86, 6.13 and 4.27 for *I*. *delicatissima*, *I*. *iguana* and hybrids respectively. All loci showed deviation of Hardy-Weinberg equilibrium for both *I*. *delicatissima* and *I*. *iguana* with excess of homozygosity (P < 0.001). 5 out of 15 loci showed significant homozygosity levels for hybrids. 11 and 59 private alleles (over a total of 111 alleles) were found for *I*. *delicatissima* and *I*. *Iguana* respectively.

### Species genetic clustering

**Fig 4:**
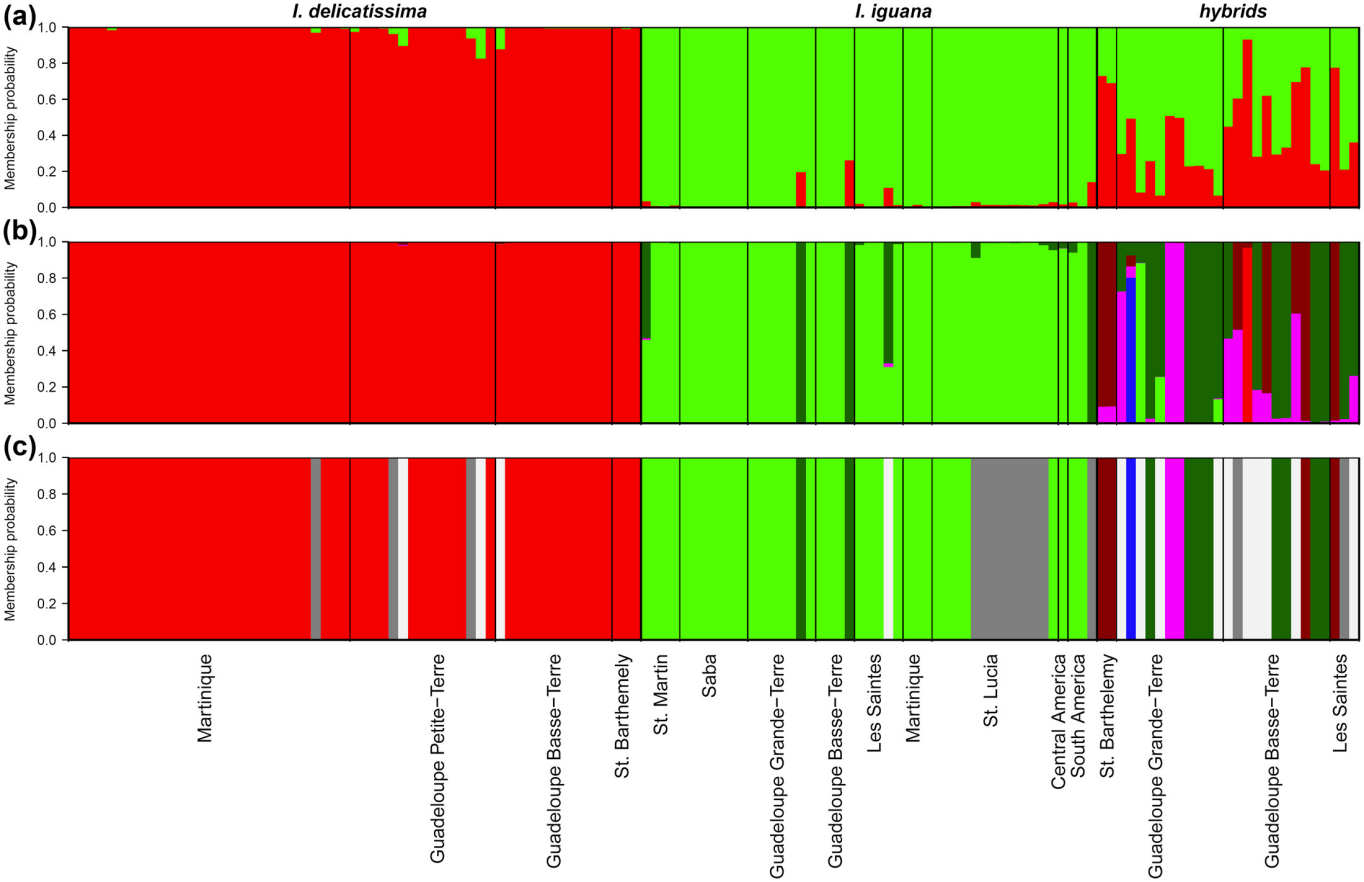
Species genetic clustering using Structure assuming two genetic clusters k = 2 (a), and hybrids assignment performed with HewHybrids (b) and HIest (c) for individuals morpholocally identified as *I*. *delicatissima*, *I*. *iguana* or hybrids. Each vertical bar represent an individual with the color indicating the membership probability to belong either to one of the two genetic cluster (a) or to one of the species or hybrid class (b and c): *I*. *delicatissima* (red), *I*. *iguana* (green), F1 (blue), F2 (magenta), *I*. *delicatissima* backcross (brown), *I*. *iguana* backcross (dark green), and for unclassified individuals resulting from the HIest analysis (c): later generation more complex hybrids (grey) and unconfident assignment (white).

Assuming two genetic clusters, Structure clearly identifies both species using genetic data only (Fig 4a, 4b and 4c). *I*. *delicatissima* individuals (N = 59) have a probability of belonging to cluster 1 ranging from 0.826 to 0.996 (mean: 0.989, sd: 0.03), while *I*. *iguana* (N = 29) have a probability of belonging to cluster 2 ranging from 0.738 to 0.996 (mean: 0.972, sd: 0.06). Morphological hybrids (N = 27) have a wide range of admixture coefficients with a probability of membership to cluster 1 ranging from 0.066 to 0.931 (mean: 0.413, sd: 0.243).

### Hybrids genetic assignment

Both NewHybrids and HIest were highly concordant with Structure in identifying species (Fig 4a, 4b and 4c). Using a relaxed criterion, NewHybrids identified 60 purebred *I*. *delicatissima*, 43 purebred *I*. *iguana*, one F1 hybrid, five F2 hybrids, five *I*. *delicatissima* backcrosses, and 19 *I*. *iguana* backcrosses (Fig 4b and Table 3). Using more stringent classification criterion, 13 individuals with less than 0.90 probability of belonging to one of the classes were left unclassified, most of them having been previously classified as *I*. *iguana* backcrosses (7) in addition to three F2, one *I*. *iguana*, one F1, and one *I*. *delicatissima* backcross (Table 3). In those uncertain cases, individual classification probabilities were intermediate between *I*. *iguana* backcross and purebred *I*. *iguana* or F2 and *I*. *iguana* backcrosses.

**Table 3:** Summary of hybrid genetic assignments and comparison of different methods and classification stringency.

	*I*. *delicatissima*	*I*. *iguana*	F1	F2	Backcrosses *I*. *delicatissima*	Backcrosses *I*. *iguana*	Unclassified	Total
NewHybrids Relax	60	43	1	5	5	19	0	133
NewHybrids Strict	60	42	0	2	4	12	13	133
HIest Best Class	59	46	1	7	6	14	0	133
HIest Strict	53	35	1	2	4	10	28^a^	133

^a^14 with two likely classes and 14 more complex hybrids

**Fig 5:**
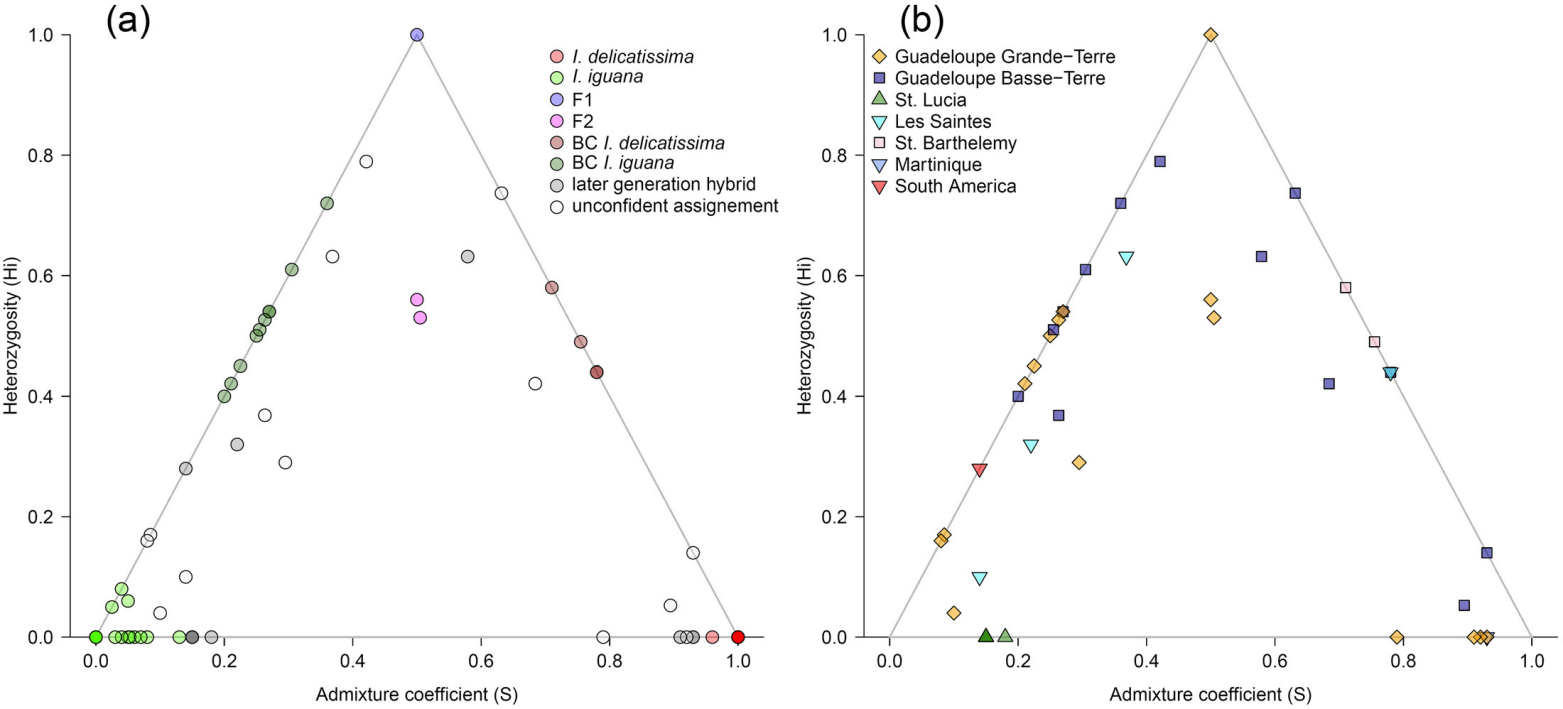
Sample space of hybrid genomic proportions. Distributions of ancestry (S) and individual heterozygosity (H_i_) on a bivariate coordinate system for all individuals according to their classification in HIest (a) and individuals assigned as hybrids only (b) identified by their population of origin.

The best class estimated for each individual using HIest is quite similar to the relaxed classification of NewHybrids (Table 3). However, using the full potential of HIest inference, a total of 28 individuals could not be classified (Fig 4c), either because their genotypes did not fit into a simple two generations hybridization model and were probably the result of a more long term admixture between species (14 individuals), or because the individual genotypes did not contain enough genetic information (due to missing data or general lack of power) in order to statistically differentiate between two likely genotypic classes (14 individuals). Interestingly, in the later cases, most individuals have been left unclassified using strict criteria in NewHybrids (10 out of 14 with only 4 classified as *I*. *delicatissima*), whereas when using relaxed criteria, individuals were classified in several different classes (4 *I*. *delicatissima*, 1 *I*. *iguana*, 3 F2, 1 *I*. *delicatissima* backcross and 6 *I*. *iguana* backcrosses). On the contrary, out of 14 individuals that were flagged as resulting from a long-term admixture, 8 were strictly assigned to *I*. *iguana*, two as *I*. *iguana* backcrosses, three as *I*. *delicatissima*, and one individual had an uncertain classification between F2 and *I*. *delicatissima* backcross. The high proportion of individuals resulting from a longer-term admixture that were classified as *I*. *iguana* or backcrosses with this species may probably indicate extensive long-term introgression of *I*. *iguana* (Fig 5a), especially in the Manganao (Grande-Terre) population (Fig 5b). Overall, the more recent method implemented in HIest provides more information about the hybridization dynamics than NewHybrids, as it allows detecting longer-term introgression while assessing the information content of the genetic data, so that the individuals can be unambiguously assigned to the different hybrid classes.

### Inter- and intra-specific genetic structure across islands

**Fig 6:**
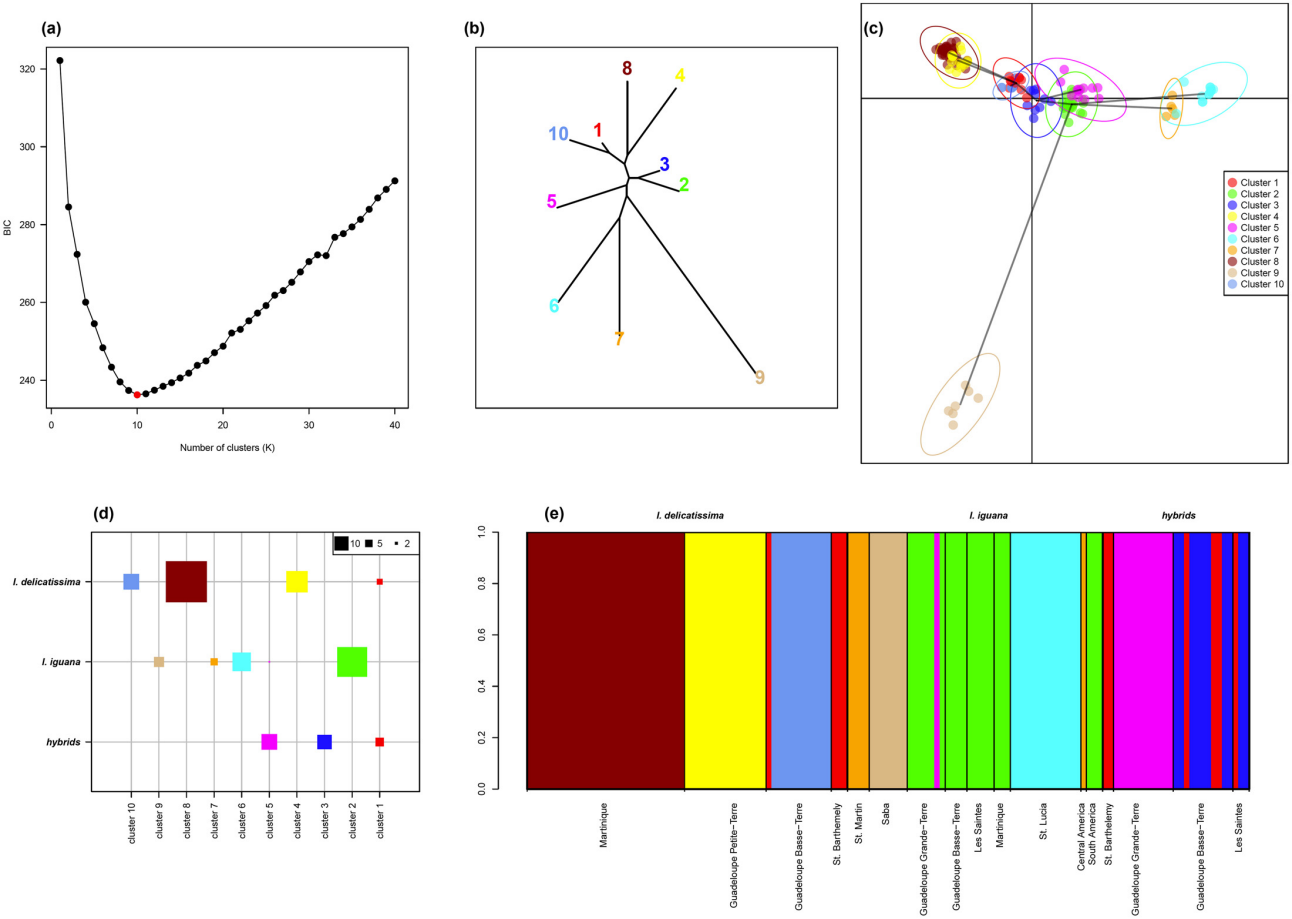
Discriminant analysis of principal component (DAPC). (a) Bayesian Information Criterion value for a range of number of cluster (K) allowed unambiguous inference of the most likely number of clusters K = 10. (b) Neighbor-joining tree computed on the DAPC distances representing the genetic relationship between the inferred 10 clusters. (c) Visualization of the location of the individuals (points) and clusters (ellipses) in the first two axes of the DAPC space (horizontal: axis 1, vertical: axis 2); a minimum spanning tree linking the two closest clusters in the entire DAPC space are represented by a grey line. (d) Distribution of the number of individual assigned to each cluster across species and hybrids. (e) Bar plot representation the individual assignment to clusters together with their species identity and population of origin.

The BIC criterion associated with the K-means algorithm clearly identified K = 10 as the most likely number of clusters to partition the genetic data (Fig 6a). The BIC value sharply decreased for K = 1 to K = 10, where it reached its minimal value and then increased for K values up to K = 40. The overall shape of the curve is in accordance with a typical hierarchical island population structure model; a case where the best number of clusters is not ambiguous and can be determined accurately [45].

*I*. *delicatissima* is represented by three specific clusters (Fig 6d and 6e: cluster 4 [Guadeloupe Petite-Terre], 8 [Martinique] and 10 [Guadeloupe Basse-Terre]), *I*. *iguana* individuals are found in four specific clusters (Fig 6d and 6e:7 [Saint Martin—Central America], 6 [St. Lucia], 2 [Grande-Terre, Basse-Terre, Les Saintes, Martinique, South America], 9 [Saba]) and hybrids are found across three clusters, one specific to hybrids (cluster 3), one shared with *I*. *delicatissima* (cluster 1) and one shared with one *I*. *iguana* individual (cluster 5). Genetic relationships between clusters clearly separated *I*. *delicatissima* (clusters 4, 8 and 10, Fig 6b) from *I*. *iguana* (clusters 6, 7, 9, Fig 6b), while clusters containing hybrids are found at mid-distance in the neighbor-joining tree (in particular clusters 5 and 3, Fig 6b). In addition, cluster 1 containing *I*. *delicatissima* and hybrids is closely related to *I*. *delicatissima* species clusters, in particular cluster 10 (Fig 6b and 6c). More surprisingly, cluster 2 containing exclusively *I*. *iguana* individuals seems to be closely related to hybrids cluster 3 (Fig 6b and 6c).

At the intra-specific level, it is noticeable that *I*. *iguana* clusters 6, 7, and 9, and *I*. *delicatissima* clusters 4 and 8, are the most distant from the hybrids clusters and show high distinctiveness (Fig 4b and 4c). These clusters are mostly found on specific islands, *i*.*e*. Martinique and Guadeloupe Grande-Terre for *I*. *delicatissima* clusters 4 and 8; St. Maarten-Martin (plus one individual from Central America), St. Lucia, and Saba for *I*. *iguana* clusters 6, 7, and 9 respectively (Fig 6e). These populations are clearly distinct at the intra-specific level and may represent historically isolated populations. Conversely, *I*. *delicatissima* cluster 10, located in Guadeloupe (Basse-Terre), shows a high genetic similarity with hybrid cluster 1, potentially indicating an ongoing and / or ancient hybridization and introgression of this population with *I*. *iguana*. *I*. *iguana* cluster 2 is found in South American populations and on several islands; notably Guadeloupe, Les Saintes and Martinique (Fig 6e). This may probably correspond to the introduced *I*. *iguana* lineage from the continent that extensively hybridized with *I*. *delicatissima* populations, thus probably explaining the close genetic proximity of this cluster with hybrids clusters (Fig 6b and 6c).

### Mitochondrial Introgression in hybrids

All 20 *I*. *delicatissima* from St. Barthélemy (Anguilla Bank), Basse-Terre and Petite Terre Guadeloupe Bank) and Chancel (Martinique) showed the same haplotype as already referenced in Genbank (AF 217786) [49]. For the 18 *I*. *iguana* analyzed, 2 individuals from Martinique and Guadeloupe (Grande-Terre) showed the haplotype specific to *I*. *delicatissima* (AF 217786). Among the 16 others, we found 5 haplotypes in our study that corresponded to haplotypes, which had already been identified by Stephen *et al*. [18] and Malone *et al*. (2000) [41] (JQ 340914 (Grande-Terre), HM 352505 (Saba), AF 217782 (St. Lucia) GB U66231 (CAM) and GB 217783 (Martinique). Out of the 18 hybrids analyzed, 10 and 8 individuals showed haplotypes respectively described in *I*. *delicatissima* and *I*. *iguana* respectively (Table 2). The comparison between both molecular markers showed that all the combinations among the different hybrids categories and *delicatissima* or *iguana* haplotypes have been observed in the data set. For example, among the three *delicatissima* backcrosses revealed with Newhybrids, two harbors *iguana* and one *delicatissma* haplotypes. In the same way, for the eight *Iguana* backcrosses obtained, six and two have *iguana* and *delicatissima* haplotypes, respectively. The haplotype recorded for *I*. *delicatissima* was identical to those described for pure individuals (AF 217786). Two haplotypes specific to *I*. *iguana* were identified corresponding to haplotypes JQ 340914 (hybrids from Grande-Terre and Basse-Terre) and AF 217782 (hybrids from St. Barthélemy and Basse-Terre).

## Discussion

### Hybridizations between both species

The results presented here provided evidence for the occurrence of hybridization between *Iguana delicatissima* and *I*. *iguana*. These phenomena had already been suspected by Breuil [50], who reported the presence of intermediate or composite phenotypes in all places where the two species are syntopic. Recently, Breuil [21] reported several morphological criteria that can characterize hybrids. Our study validated the use of these criteria to characterize hybrids since all the iguanas identified as hybrids show hybrid genotypes. However, the precise identification of specimens could be inefficient, due to the complex pattern of admixture inferred from microsatellites. Then, few specimens considered as belonging to parental species showed a low level of introgression. Day & Thorpe [25] reported that a combined approach, using both morphological and genetic criteria, could be very efficient in detecting hybridization in *Iguana*.

It is here demonstrated that hybrids are able to reproduce with parental species and between them. From our data set, the analyses revealed the existence of F1, F2, and backcrosses from both species, with mitochondrial introgression in some cases. Such fertile hybrids had already been reported by Gutsche & Köhler [8] between two other iguanids species *Ctenosaura similis* and *C*. *bakeri*, which have different habitats. Hybrids fertility yields to introgression and genetic absorption. This evidence of fertile hybrids is supported by introgression of mtDNA from one species to another. For example, two specimens from Fort-de-France having a nuclear genotype characteristic of *I*. *iguana* and classified as pure species according to morphological traits, had a mitochondrial haplotype specific from *I*. *delicatissima*.

Our results indicate that natural hybridization occurs in both directions because mitochondrial introgression has been observed in hybrids, and also in both morphologically pure individuals. According to DAPC analysis, the hybrids are grouped in three clusters (1, 3, 5). Cluster 1 groups *I*. *delicatissima* and hybrids from St. Barthélemy, Basse-Terre, and Terre-de-Haut des Saintes. It has already been shown in this study that *I*. *delicatissima* from St. Barthélemy is closer to *I*. *delicatissima* from Basse-Terre than those from Grande-Terre and, as a consequence, their hybrids share the same *delicatissima* contribution. It is noteworthy that one common iguana from Les Saintes belongs to this cluster. This suggests that the lost *I*. *delicatissima* from Les Saintes is close to *I*. *delicatissima* from Basse-Terre, whereas these islands are not located on the same bank. Cluster 3 contains only hybrids from Basse-Terre and Les Saintes. This cluster is close to the common iguana from South America, which is at the origin of all populations in Les Saintes, Basse-Terre, Grande-Terre, and Martinique. This is one more argument that suggests that *I*. *iguana*, which hybridize on these islands, are from South America. Cluster 5 contains only iguanas from Grande-Terre. The *delicatissima* genetic contribution of these hybrids is close to the genetic pool of Petite Terre, according to the proximity of these two islands that belong to the same bank. One specimen of Grande-Terre considered morphologically as *I*. *iguana* falls into this cluster. Such a situation reveals the difficulties of assigning some individuals to a precise category when the level of admixture is too high.

The level of hybridization seems to differ across places. For example, in Grande-Terre, most hybrids are considered to be the result of backcrossing with *I*. *Iguana* and the other ones as F2 with respect to microsatellite analysis. When Breuil [50, 51, 52] discovered this population in a disturbed area, only one female and one male *I*. *delicatissima* were observed in this place, whereas all the other iguanas were phenotypically hybrids and *I*. *iguana*. This situation can be explained by the existence of a very small-sized relictual *I*. *delicatissima* population that was invaded by numerous common iguanas progressing eastward from the harbour of Pointe-à-Pitre where they arrived during the 1980s-1990s.

This is the opposite of St Barthélemy, where hybrids were assigned to backcrossing with *I*. *delicatissima*. This situation is due to the fact that only some common iguanas from St Maarten arrived at the beginning of the 21^st^ century and that there still are hundreds of *I*. *delicatissima* on this island. Therefore, the probability for a hybrid to reproduce is higher with a *I*. *delicatissima* than with a common iguana. Basse-Terre showed an intermediate situation with backcrossing from both species, but mainly from *I*. *iguana*. Historical data indicate that the first common iguanas arrived from Les Saintes in South Basse-Terre, where they began to proliferate at the end of the 1950s. The data also reveal that they were also present at the beginning of the 1960s near the localities (UCPA—Anse à Sable) where we have both backcrosses. At the beginning of the 1990s, *I*. *iguana* and hybrids were common in the region of Carangaise-Longueteau. In this locality, there are mainly backcrosses with the common iguana and F2. The last region to be invaded by *I*. *iguana* was North Basse-Terre (Cluny) where no common iguana was present at the beginning of the 21^st^ century [19, 21]. The hybrids of this locality are F2 and the two backcrosses suggest that, at the time of sampling (2007–2012), no parental species outnumbered the other. However, as iguanas are animals with a long life expectancy, this could suggest that the first hybridization produced offspring that backcrossed between them and with *delicatissima* that outnumbered the common iguana at the beginning of the colonization of that place. In the whole Basse-Terre, only few *I*. *delicatissima* individuals were still present. This species may live more than 20 years, the last remaining females are mainly fertilized by F1 or F2 hybrids and by more or less introgressed *I*. *iguana*.

According to the DACP analysis, *I*. *iguana* sampled on Les Saintes, Basse-Terre, Grande-Terre and Martinique, cluster with specimens from Venezuela and French Guyana. This result accords with the work of Lazell [22], who reported that the common iguanas he observed on Les Saintes were morphologically identical to those of Northern South America. Our genetic analysis clearly demonstrates that the common iguanas found in les Saintes, Basse-Terre, and Grande-Terre, were introduced from Northern South America (See review in Breuil) [12, 19, 21] and are not a cryptic taxon as suggested in Guadeloupe. The common iguanas from Central America and South America constitute an invasive lineage in numerous regions: Florida [53], the Dominican Republic [54], Puerto Rico [55], St. Lucia [56], and numerous islands in the Pacific [57].

### Causes and consequences of genetic admixture

Allendorf *et al*. [58] distinguished natural hybridization from anthropogenic hybridization. Anthropogenic hybridization occurs when human activities are directly or indirectly responsible for the contact between both taxa. Anthropogenic hybridization falls into three groups: hybridization without introgression when F1 hybrids are sterile, hybridization with widespread introgression, and complete admixture when F1 hybrids are fertile. It is clear from our results that iguanas fall into this third group. For example in Les Saintes, there are no more *I*. *delicatissima*, only *I*. *iguana* with some *delicatissima* haplotypes. In Grande-Terre, where *I*. *delicatissima* has not been observed recently, *I*. *iguana* are very abundant and some of them could be introgressed. Similar cases of genetic pollution due to human transfers have been reported on the population of West Indian slider turtles (*Trachemys* genus) [59].

In our case, *I*. *iguana* outcompetes *I*. *delicatissima* when in the same location. *Iguana iguana* males are more powerful than *I*. *delicatissima ones*. Therefore, they are able to displace them and to reproduce with *I*. *delicatissima* females (Breuil pers. obs.) [13]. We do not know anything about the hybrids’ behavior or how they choose their mates. Nevertheless, F1, F2, and *iguana* backcrosses are bigger and longer than even older *I*. *delicatissima* males. So we believe that they are also able to displace the endemic species and to reproduce with different kinds of females.

Moreover, *I*. *iguana* reproduces about 1.5 months earlier in the season, which, after a three-month incubation period, yields to hatchlings in mid-August. Thus new-born common iguanas have a longer growth period, which allows them to reach a greater size for their first dry season and increases their survival rate. Moreover, *I*. *iguana* young males could be sexually active before the *I*. *delicatissima*, thus copulating with *delicatissima* females without facing any sexual competition. We roughly estimate that *I*. *iguana* lay two to three times more eggs than *delicatissima* [12] and that colonization by the new invader is very efficient. Consequently, in place where *I*. *iguana* have been present for a long period of time, the species replaces *I*. *delicatissima* through hybridization.

Over the last 50 years, we have lost *I*. *delicatissima* from Les Saintes (at least 2 islands) and Grande-Terre through genetic admixture. In Basse-Terre, the phenomenon is ongoing and youngs *I*. *delicatissima* are very rare. In St. Barthélemy, the phenomenon has been initiated. We have no data for St. Maarten-Martin, but *delicatissima* must have disappeared before the arrival of *I*. *iguana* from different origins. In Martinique, *I*. *iguana* is in close contact with the populations of Islet Chancel and Northern Martinique, but they are not known to be sympatric [15]. *I*. *iguana* may arrive soon in La Désirade, just as it has on Marie-Galante and St. Maarten-Martin these very last years [13, 24, 29]. The same threat holds for the population of Petite Terre, but its Natural Reserve status and the presence of wardens who are aware of this risk may prevent such an invasion. However, an isolated male can unnoticeably arrive and copulate with several females in a remote part of the islands. For inexperienced people, it is nearly impossible to identify a new-born individual. Thus, we may have dozens of F1 individuals that will be able to reproduce two years from now. In this situation, the population will quickly lose its specificity through admixture.

### Saving the last *I*. *delicatissima* in Basse-Terre

If we want to save the last *I*. *delicatissima* in Basse-Terre, we have to catch the phenotypically pure *I*. *delicatissima*, using the chart of Breuil [12, 21] and our genetic analysis. During these careful investigations, iguanas should be held in captivity. Pure *delicatissima* should be used for translocation programs in selected islets, such as Islet Kahouanne, offshore of North Basse-Terre, where *I*. *delicatissima* were previously known [12], or in Marie-Galante [29] as on other islets from other Lesser Antillean states. Some of these pure *I*. *delicatissima* could also be used for breeding programs in French zoos, including in Martinique and Guadeloupe, as well as in other zoos abroad. In captivity, it will also be possible to monitor the breeding behavior of the different types of iguanas and to estimate the fertility of the different mating types. Although *I*. *delicatissima* has been held in captivity in different institutions, reproduction is very difficult to obtain [14].

In populations where the numbers of *I*. *iguana* and hybrids are low, while *I*. *delicatissima* is quite abundant, such as in St. Barthélemy, and where there is no common iguana population in the vicinity, an attempt could be made to withdraw hybrids and common iguana, so as to restore a pure *I*. *delicatissima* population *in situ*. This proposal requires numerous observations and the identification of all the iguanas present in such a locality, in order to be sure to remove all unpure *delicatissima* iguanas.

*I*. *iguana* has been fully protected in Guadeloupe since 1989 because, at that time and based on Lazell’s work, this species was thought to be autochthonous. On the contrary, it is not protected in Martinique, as it was known that it was introduced there [12]. Since 2005, wardens from Office National de la Chasse et de la Faune Sauvage have been allowed to catch them in Martinique. In Guadeloupe, despite the request of the Ministry of Ecology in 2006 to withdraw *I*. *iguana* from the protected list, it took 8 years to see that text published. As of 2014, *I*. *iguana* is no longer protected in Guadeloupe and St Martin, but nothing is done to control *I*. *iguana* populations, even in the most critical situations.

Thus, saving *I*. *delicatissima* on Basse-Terre and preventing an *I*. *iguana* invasion of other islands will be very difficult and costly with uncertain results [13, 24]. Since the 1960s, *I*. *delicatissima* has been eliminated from at least 7 islands and islets of FWI and *I*. *iguana* has arrived on three or four islands since the 1990s (Fig 1). The 2012 discovery of a new born *I*. *iguana* on Islet Kahouanne and the inability of local administrations, such as Parc National de la Guadeloupe, the local Direction of Environment of Guadeloupe, the municipality of Deshaies, which Islet Kahouanne belongs to, and the local association that prepared this translocation, to agree to this project, has led to its resignation. In this condition, perhaps the only solution to keep *I*. *delicatissima* on Basse-Terre is to build a breeding farm, as it has been the case for *I*. *iguana* in Central America. Moreover, an adult *I*. *iguana* was observed by the NGO Karisko in November 2013 on Ilet Ramiers (Martinique), where 9 *I*. *delicatissima* were translocated in 2006 [15].

Finally, there is an ongoing large study on genetic variability from pure *I*. *delicatissima* populations in French West Indies, which goals are to assess the originality of *I*. *delicatissima* for conservation programs on each island, and to translocate individuals according to their genetic proximity, which does not always match their geographical locations. The demonstration of hybrids’ fertility and introgression between both species is very important with respect to conservation issues and translocation programs [13, 14]. In a near future, it will be necessary to study the genetic structure from microsatellites in Dominica, Anguilla, and Statia, but also to increase our samples for the FWI islands, in order to better understand the relation between these different populations and the action of Amerindians with respect to the use of iguanas.

## Supporting Information

S1 Table Multilocus microsatellite genotyping. (XLS)
